# 670. Should Serial Casting be a “First-Resort” Conservative Treatment for Adults with Upper Extremity Burn Contractures?

**DOI:** 10.1093/jbcr/irag033.551

**Published:** 2026-04-06

**Authors:** Zoë Edger-Lacoursière, Valérie Calva, Victorine Labonté, Ingrid Malo-Leclerc, Geneviève Schneider, Ariane Vaillancourt, Stéphanie Jean, Elisabeth Marois-Pagé, Bernadette Nedelec

**Affiliations:** Villa Medica Rehabilitation Hospital and McGill University, Montréal, QC; Villa Medica Rehabilitation Hospital, Montréal, QC; Villa Medica Rehabilitation Hospital, Montréal, QC; Villa Medica Rehabilitation Hospital, Montréal, QC; Villa Medica Rehabilitation Hospital, Montréal, QC; Villa Medica Rehabilitation Hospital, Montréal, QC; Université de Montréal, Montréal, QC; Villa Medica Rehabilitation Hospital, Montréal, QC; McGill University, Montréal, QC

## Abstract

**Introduction:**

Serial casting is commonly used as a last resort treatment when patients do not respond to traditional therapy or are not adherent to prescribed treatment. However, studies have demonstrated that casting provides wound protection, and maintenance or improvement joint mobility. In addition, based on recent mechanotransduction literature, it is possible that plaster casts have post-burn scar management benefits. Thus, the aim of this study was to evaluate changes in range of motion (ROM), scar characteristics, and patient-reported upper-extremity (UE) function following an individualized serial casting program compared to usual care. Follow up data was collected 3 weeks after casting was discontinued to assess whether changes were maintained.

**Methods:**

UE joints with >15% loss of passive ROM first received usual care for at least 3 days before starting serial casting. Passive and active ROM, scar thickness, elasticity, erythema, transepidermal water loss, and melanin were measured at baseline, twice weekly during casting, and 3 weeks post-treatment. Patient-reported UE function and scar satisfaction were assessed at baseline and 3 weeks post-treatment using the QuickDASH and Patient Scar Assessment Scale (PSAS). For each participant, ROM and scar data were plotted over time, with a regression line derived from the two pre-cast measurements to represent the “best-case scenario” for usual care. Casting was deemed more effective when greater than half the casting to 3w post measures exceeded this projection. Binomial calculations determined the study’s required sample size (n = 12) and the minimum number of successes required for statistical significance.

**Results:**

Ten individuals (13 joints) were included in this study. Eleven (11/12) casted joints achieved regular PROM (1 participant was removed due to missing data). Revised ROM could not be calculated for MCP joints, thus 8/8 revised PROM and 8/8 revised AROM above the projected usual care, all of which were statistically significant. Scar thickness was the only scar characteristic that significantly improved based on the projected line for 11/13 joint measures. QuickDASH improved for 11/13 joints (median 16%, 20.45-9.18) and PSAS improved for all joints (median 6, IQR 4-10).

**Conclusions:**

The data demonstrated that serial casting is effective for reducing contractures and HSc thickness, and improving patient-perceived UE function and scar outcome.

**Applicability of Research to Practice:**

This study is the first to investigate the effects of plaster casts on HSc using objective measures. Clinicians can confidently use serial casting to treat post-burn scar contractures and HSc thickness.

**Funding for the study:**

Foundation funding.

